# Molecular and pathological insights into gene expression and oxidative stress in *Clinostomum complanatum* and *Euclinostomum heterostomum*

**DOI:** 10.1038/s41598-025-16469-5

**Published:** 2025-10-28

**Authors:** Mai A. Salem, Olfat A. Mahdy, Mohamed A. El-Saied, Mohamed S. Kamel, Faten F. Mohammed, Reem M. Ramadan

**Affiliations:** 1 Department of Parasitology, Faculty of Veterinary Medicine, Cairo University, Giza, Egypt; 2 Department of Pathology, Faculty of Veterinary Medicine, Cairo University, Giza, 12211 Egypt; 3 Department of Medicine and Infectious Diseases, Faculty of Veterinary Medicine, Cairo University, Giza, 12211 Egypt; 4 Department of Pathology, College of Veterinary Medicine, King Faisal University, 31982 Al-Ahsa, Saudi Arabia

**Keywords:** *Clinostomum complanatum*, *Euclinostomum heterostomum*, PCR, Gene expression, Oxidative stress, Immunohistochemistry., Immunology, Zoology

## Abstract

Parasitic infections caused by *Clinostomum complanatum* and *Euclinostomum heterostomum* pose significant threats to aquaculture systems and public health. This study examined their molecular, immunological, and pathological impacts in Egyptian Nile tilapia (*Oreochromis niloticus*), with COXI gene sequencing confirming both species and revealing genetic similarities to regional isolates, suggesting broad geographical distribution. *Euclinostomum heterostomum*-infected fish exhibited significant upregulation of immune-related genes (*IFN-γ*: 1.96-fold; *IL-10*: 2.06-fold; *IL-12*: 1.91-fold; *IL-1β*: 4.07-fold; *CYP-1α*: 2.07-fold) and elevated oxidative stress markers (SOD: 2.96-fold; CAT: 3.52-fold; GSH: 3.07-fold; TAC: 2.58-fold) compared to uninfected controls (*p* < 0.0001 for all comparisons). Similarly, *Clinostomum complanatum* infections triggered upregulation of *IFN-γ* (2.17-fold), *IL-10* (2.09-fold), *IL-12* (2.25-fold), *IL-1β* (2.51-fold), and *CYP-1α* (2.58-fold), alongside increased SOD (2.63-fold), CAT (2.70-fold), GSH (2.99-fold), and TAC (2.65-fold) compared to uninfected controls (*p* < 0.0001 for all comparisons). Histopathology revealed necrosis, fibrosis, and inflammation in gill and kidney tissues, with immunohistochemical staining confirming localized inflammatory markers. These findings demonstrate systemic immune activation and physiological stress caused by clinostomid infections, providing critical insights for aquaculture disease management strategies.

**Supplementary Information:**

The online version contains supplementary material available at 10.1038/s41598-025-16469-5.

## Introduction

Clinostomids, particularly *Clinostomum complanatum* (*C. complanatum*) and *Euclinostomum heterostomum* (*E. heterostomum*), are parasitic trematodes of significant concern due to their complex life cycles and impacts on aquatic ecosystems, fish health, and occasionally, human health. These parasites utilize snails as the first intermediate hosts, fish as the second intermediate hosts, and fish-eating birds as definitive hosts^1^. Infected fish often exhibit severe tissue damage, making these parasites a challenge for aquaculture and fishery management systems. Grossly, infected fish often exhibit visible cysts on the gills, skin, and musculature, which can cause deformities, impaired movement, and economic losses due to decreased marketability^2^.

Although the parasitic burden caused by Clinostomids is well-documented, there is still limited understanding of the host’s molecular and immunological responses to these infections, particularly in economically important species such as *Oreochromis niloticus (O. niloticus)*. Addressing this knowledge gap is essential for developing effective disease management strategies in aquaculture systems.

Advancements in molecular biology have enabled the analysis of gene expression in hosts infected by parasites^1^. Studies have revealed the upregulation of immune-related genes and antioxidant enzymes in response to trematode infections, reflecting the host’s efforts to counteract parasitic invasion^3^. We hypothesized that natural infection by *Clinostomum complanatum* and *Euclinostomum heterostomum* elicits distinct immune and oxidative stress responses in the affected tissues of Nile tilapia. Immunohistochemical analyses have further demonstrated localized immune responses in infected tissues, with increased activity of markers such as lysozyme and alkaline phosphatase in response to parasitic antigens. These immune responses involve both innate and adaptive mechanisms, characterized by inflammation and immune cell infiltration at the infection sites^4^.

The roles of various immunological cytokines, including interferon-gamma (*IFN-γ*), interleukin-10 (*IL-10*), interleukin-12 (*IL-12*), cytochrome P-450 1α (*CYP-1α*), and interleukin-1 beta (*IL-1β*) in fish infected with pathogens have been explored in some studies^5^. These cytokines (IFN-γ, IL-10, IL-12, IL-1β) are pro-inflammatory mediators, critical during the early stages of infection^6^. Pro-inflammatory cytokines also play a crucial role in limiting parasite growth and promoting chronic latent infections through cyst formation^7^. Despite their importance, there remains a lack of studies evaluating the immunological condition of *O. niloticus* infected with *Clinostomum* spp., particularly in Egypt.

Parasitic infections trigger oxidative stress as hosts generate reactive oxygen species (ROS) to counter pathogens^8^. However, ROS can harm both host cells and parasites, leading to oxidative damage in DNA, proteins, and lipids, which are vital for cellular function. DNA damage may cause mutations and genomic instability, lipid peroxidation can weaken membrane integrity, and protein oxidation can disrupt metabolic processes^9^.

Parasites have well-documented pathological effects on fish, including necrosis, hemorrhage, and fibrosis in tissues affected by metacercariae^10^. Oxidative stress plays a significant role in host-parasite interactions, occurring when ROS production exceeds the host’s antioxidant capacity, resulting in cellular damage and inflammation^11^. Research has shown that trematode-infected fish experience increased oxidative stress, leading to lipid peroxidation and weakened tissue structures^2^.

The present study aims to provide comprehensive molecular and pathological insights into *C. complanatum* and *E. heterostomum* infections in *O. niloticus*. It investigates the immunological response through quantitative real-time PCR (qRT-PCR) analysis of *IFN-γ*,* IL-10*,* CYP-1α*,* IL-1β*, and *IL-12* gene expression, alongside molecular identification, assessment of oxidative stress markers, histopathological alterations, and immunohistochemical responses of tumor necrosis factor-alpha (*TNF-α*), interleukin-6 (*IL-6*), caspase-3, and inducible nitric oxide synthase (iNOS). These integrated findings are intended to enhance the understanding of host–parasite interactions and support the development of effective control strategies in aquaculture systems.

## Materials and methods

### Ethical approval

This study was conducted in accordance with the ethical guidelines set by the Institutional Animal Care and Use Committee (IACUC) of the Faculty of Veterinary Medicine, Cairo University. All methods are reported per ARRIVE guidelines (https://arriveguidelines.org).

### Fish sampling

The present study took place between April and October 2024. Researchers collected 250 Nile tilapia (*O. niloticus*) from the Nile River in Giza Governorate (29°59’13.2” N, 31°12’42.5” E) to examine clinostomid infections. The fish, weighing between 100 and 199 g, were sourced from water drains. Fishermen reported spotting unusual yellow grubs in the branchial cavity and on the skin. They captured the fish and transported them alive in oxygenated plastic bags to the Parasitology Laboratory at the Faculty of Veterinary Medicine, Cairo University. Upon arrival, the fish were placed in aerated aquaria filled with dechlorinated tap water to stabilize them before further examination.

### Parasitological examination

Fish were euthanized using an overdose of MS-222 (Sigma) before undergoing examination for parasitic infections following standard diagnostic procedures. The methodology outlined by Mahdy et al.^10^ was applied, involving direct observation, a hand lens, and a stereo microscope (Olympus-SZ20). Metacercariae were isolated from their cysts employing a sharp needle and subsequently washed in physiological saline solution in Petri dishes. The parasites underwent preservation between glass slides using 70% ethanol. They were then stained with acetic acid, alum, and carmine, followed by dehydration through a series of alcohol concentrations. Finally, the specimens were cleared using clove oil and mounted in Canada balsam to facilitate microscopic examination^12^. The prevalence of clinostomid infection was calculated using the standard epidemiological formula: Prevalence (%) = (Number of infected fish / Total number of examined fish) × 100.

### Molecular identification

Ten worm specimens from each species were cleaned, rinsed with 1% sterile saline, and stored at -20 °C in Eppendorf tubes. DNA extraction from cysts was carried out using the QIAamp DNA Mini Kit (Qiagen, Germany) following the tissue protocol, and the extracted DNA’s purity and concentration were assessed using a Nanodrop2000 spectrophotometer (NP80, Nanophotometer, Implen, Germany).

PCR amplification targeted the cytochrome oxidase subunit I (COXI) gene using degenerate primers: MplatCOXIdF (5′-TGTAAAACGACGGCCAGTTTWCITTRGATCATAAG-3′) and MplatCOXIdR (5′-CAGGAAACAGCTATGACTGAAAYAAYAIIGGATCICCACC-3′), as designed by Moszczynska et al.^13^. Each PCR reaction, conducted in a 25 µL volume, utilized the Emerald Amp Max PCR Master Mix (Takara, Japan) with slight modifications to thermal cycling conditions. The protocol included an initial denaturation at 94 °C for 2 min, followed by 35 cycles of denaturation at 94 °C for 30 s, annealing at 50 °C for 30 s, extension at 72 °C for 1 min, and a final extension at 72 °C for 10 min^14,15^.

The PCR products were purified using the QIAquick PCR Purification Kit (Qiagen, USA) and sent to Macrogen Inc. (Seoul, South Korea) for bidirectional sequencing using the same primer pairs. Sequencing was carried out with the Big Dye Terminator Cycle Sequencing Kit on an Applied Biosystems™ 3730XL sequencer. Raw sequencing data were edited and assembled using BioEdit software 7.7. Prior to phylogenetic tree construction, the NCBI BLAST tool was used to compare the obtained sequences with existing entries in the database, enabling the selection of closely related sequences for tree analysis^16^. The resulting sequences were then submitted to GenBank to obtain accession numbers. Phylogenetic analysis was conducted using MEGA 11 software, employing the maximum likelihood method using Jukes-Cantor substituion model with 1,000 bootstrap replicates. A matrix table detailing phylogenetic identity and genetic distance was constructed based on multiple alignments and pairwise comparisons using the maximum likelihood model.

### Analysis of pro-inflammatory cytokines by quantitative real-time PCR (qRT-PCR)

Samples were collected from 20 heavily infected fish, each exhibiting an infection intensity of 10–12 encysted metacercariae (EMCs). Pharyngeal tissues and gills infected with *Clinostomum* spp., as well as kidneys infected with *Euclinostomum* EMC, were aseptically dissected. Additionally, samples from five uninfected fish, maintained in a controlled laboratory aquarium and thoroughly examined to confirm the absence of parasites, were used as negative controls. All samples were aseptically stored at -20 °C. The collected samples were categorized based on the parasite type, distinguishing those infected with *Clinostomum* from those infected with *Euclinostomum*.

The total RNA from 100 mg samples of gill, pharynx, and kidney was isolated using the Ambion Total RNA Kit (Applied Biosystems) according to the supplier’s instructions. Briefly, tissue homogenization was performed in D tubes with a Lysing Matrix (MP Biomedicals) in two cycles of 30 s at a speed of 6 m/s using a FastPrep 24 homogenizer (MP Biomedicals). The concentration and purity of the RNA were determined using a Thermo Scientific Nanodrop spectrophotometer. An RNA sample of 500 ng was treated with DNase I (Invitrogen) for amplification-grade treatment as per the manufacturer’s guidelines. Subsequently, we reverse-transcribed the treated RNA into complementary DNA (cDNA) using the High-Capacity cDNA Archive Kit (Applied Biosystems)^6^.

Primer sets specific to *O. niloticus* for IFN-γ, IL-10, IL-12, IL-1β, and CYP-1α were designed based on previously published sequences available in GenBank (Table 1). β-actin was used as the reference gene for normalization. Gene expression was assessed using cDNA pools derived from ten previously screened, parasite-free fish. Additionally, five non-infected control fish, which were carefully examined to confirm parasite absence, were used for further gene expression analysis^17^. qRT-PCR data were analyzed using the 2^-ΔΔCt method with β-actin as the housekeeping gene. Primer efficiency ranged from 95 to 105%, validated through standard curves. Data represent three biological replicates, each with three technical replicates. The qRT-PCR amplification was performed for 40 cycles with the following parameters: denaturation at 94 °C for 30 s, annealing at 60 °C for 30 s, and extension at 72 °C for 45 s. The real-time PCR protocol followed the methodology outlined by Salem et al.^18^.

**Table 1:** Sequences of oligonucleotide primer for qRT-PCR.

Genes	Primers sequences	Accession number	References
*IFN-γ*	F-TCAACCCCTTCTCGCCACT R-GCTGCCTACTTGGTCCCTGA	AM261214	^19^
*IL-1B*	F-GCTGGAGAGTGCTGTGGAA R-GAACCTGGAGCATCATGGCG	X54796.1	^20^
*IL-10*	F-AGAACCACGACCCAGACATC R-CCACCGCCTTGCTCTTATTC	JX976621	
*IL-12*	F-CCTCCTCAGAGCAAAGTG R-CCCAGCACAAACAAAGAC	AM944367	^21^
*CYP-1α*	F-ATGACACGCTGGAGGACTTC R-TGGCTGGAGTGGCTGAAA	FJ389918	
*β-actin*	F-GGCTACTCCTTCACCACCACAG R-GGGCAACGGAACCTCTCATT	KJ126772.1	

### Measurements of oxidative stressor biomarkers

The study evaluated oxidative stress markers and cytokine expression levels in different experimental groups. Serum samples from both infected and non-infected fish were analyzed for catalase (CAT), glutathione (GSH), superoxide dismutase (SOD), and total antioxidant capacity (TAC) using specialized diagnostic kits^22^.

### Histopathological and immunohistochemical examination

For histopathological analysis, gill and kidney tissues from infected fish were fixed in buffered neutral formalin^23,24^sectioned, and examined under an Olympus BX43 light microscope. Images were captured using an Olympus DP27 camera with cellSens dimensions software. The immunohistochemical analysis involved processing tissue sections on positively charged slides (5 μm thick), rehydrated with phosphate-buffered saline. Monoclonal antibodies targeting TNF-α, IL-6, Caspase-3, and iNOS (sc-52746, sc-130326; Santa Cruz Biotechnology, Inc., Heidelberg, Germany) were used, following Taha et al.^25^ methodology. Briefly, histological sections were cleared in xylene, rehydrated in descending alcohol concentrations, and washed with phosphate buffer saline (PBS) at pH 7.4 for 5 min. Antigen retrieval was done using microwave after immersion in Tris/EDTA buffer (pH 9.0) for 5 min. Blocking of endogenous peroxidase by incubation with H2O2 for 5 min, and bovine serum was added for 30 min. incubation with primary antibodies, IL-6, Caspase-3, and iNOS (dilution,1:200) for 1 h, washing with PBS was performed, followed by incubation with HRP-labeled secondary antibody and DAB-substrate kit (Bio SB, USA), and counterstained with Mayer’s hematoxylin, was applied.

After dehydration, sections were mounted and images were captured with Cell Sens Dimension software, Olympus, Tokyo, Japan. Negative control slides were prepared by removing the primary antibody incubation step.

### Statistical analysis

Statistical tests were conducted using IBM SPSS Statistics version 26 and R software to compare the infected and control fish groups (*n* = 5 per group)^26,27^. The normality of the data was first assessed using the Shapiro-Wilk test. To evaluate significant differences between the groups, a student’s t-test was employed^28^. A significance level of 0.05 was adopted, with results marked as significant at *p* < 0.05 (*), *p* < 0.01 (**), *p* < 0.001 (***), and *p* < 0.0001 (****).

## Results

### Prevalence of infection with larval stages of Clinostomids

**Fig. 1:**
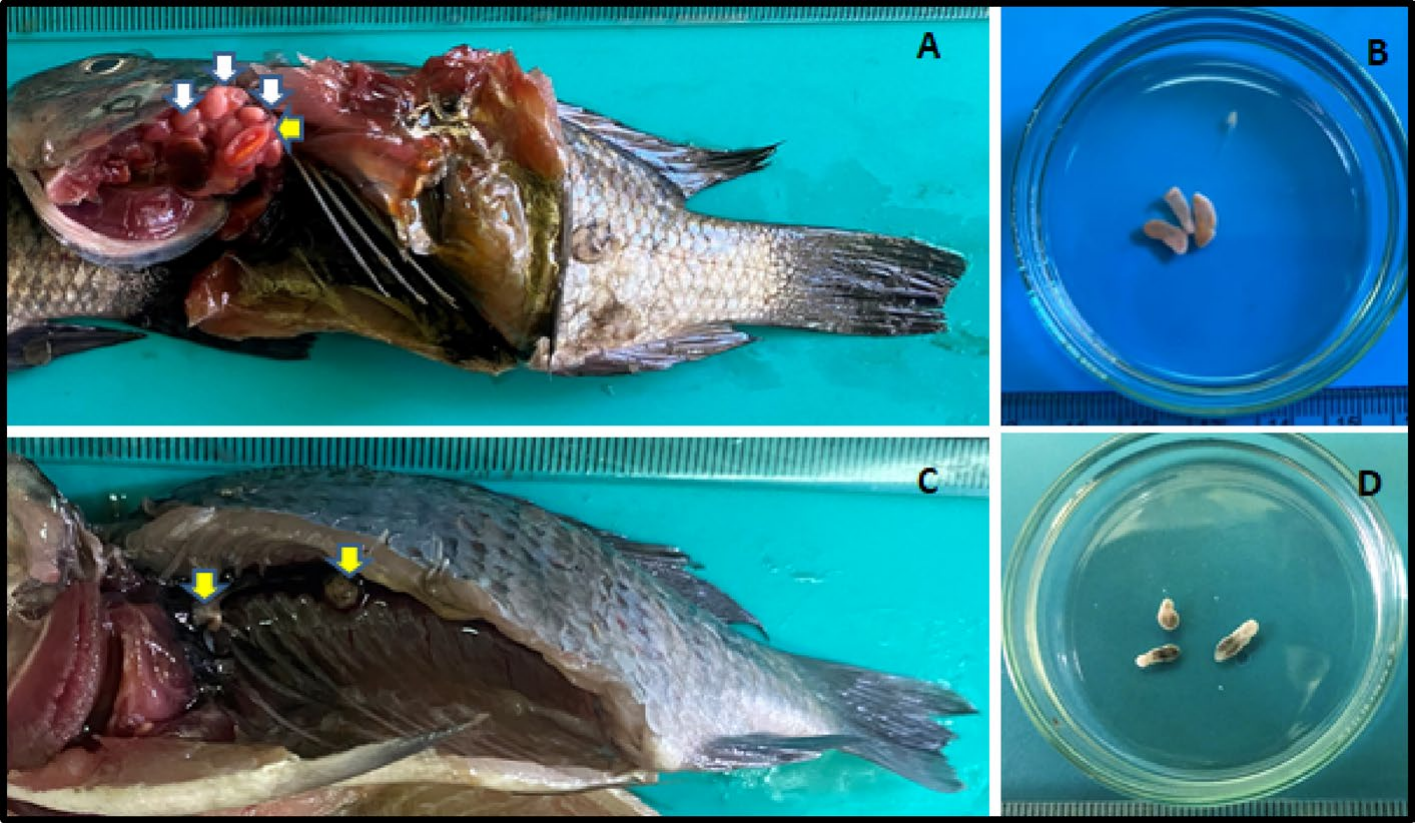
(**A**) *O. niloticus* infected with *C. complanatum*, showing small yellowish cysts in the buccal cavity (white arrows), Excysted EMC yellow arrow. (**B**) Excysted metacercariae of *C. complanatum*. (**C**) *E. heterostomum* EMC in the kidney, presenting as greyish, spherical cysts in the peritoneum surrounding the kidney (arrows). (**D**) Excysted metacercariae of *E. heterostomum*.

The present study identified infections in Nile tilapia (*O. niloticus*), involving multiple cyst types, primarily *Clinostomum* and *Euclinostomum* species. A total of 250 randomly selected fish were examined for fish-borne trematodes in the Giza Governorate. The overall infection prevalence was 42.8%, with 107 out of 250 fish testing positive for external metacercariae (EMC). *Clinostomum species* were primarily found in the buccal cavity, followed by the gill arches. *Euclinostomum* metacercariae were predominantly located in the kidneys. The cysts of *Clinostomum* were firmly embedded in tissues, surrounded by a thick capsule, and varied in size, with larger cysts measuring 6.0–9.5 mm and smaller ones measuring 0.8–3.5 mm (Fig. 1A and B). *Euclinostomum* EMC appeared pea-shaped and pale white, while *Clinostomum* EMC exhibited a yellow to yellowish-white, grape-like appearance. Juvenile worms of both species were highly motile, displaying strong elasticity and contractility (Fig. 1C and D).

### Molecular identification

#### Phylogenetic analysis of Clinostomids sequences

**Fig. 2:**
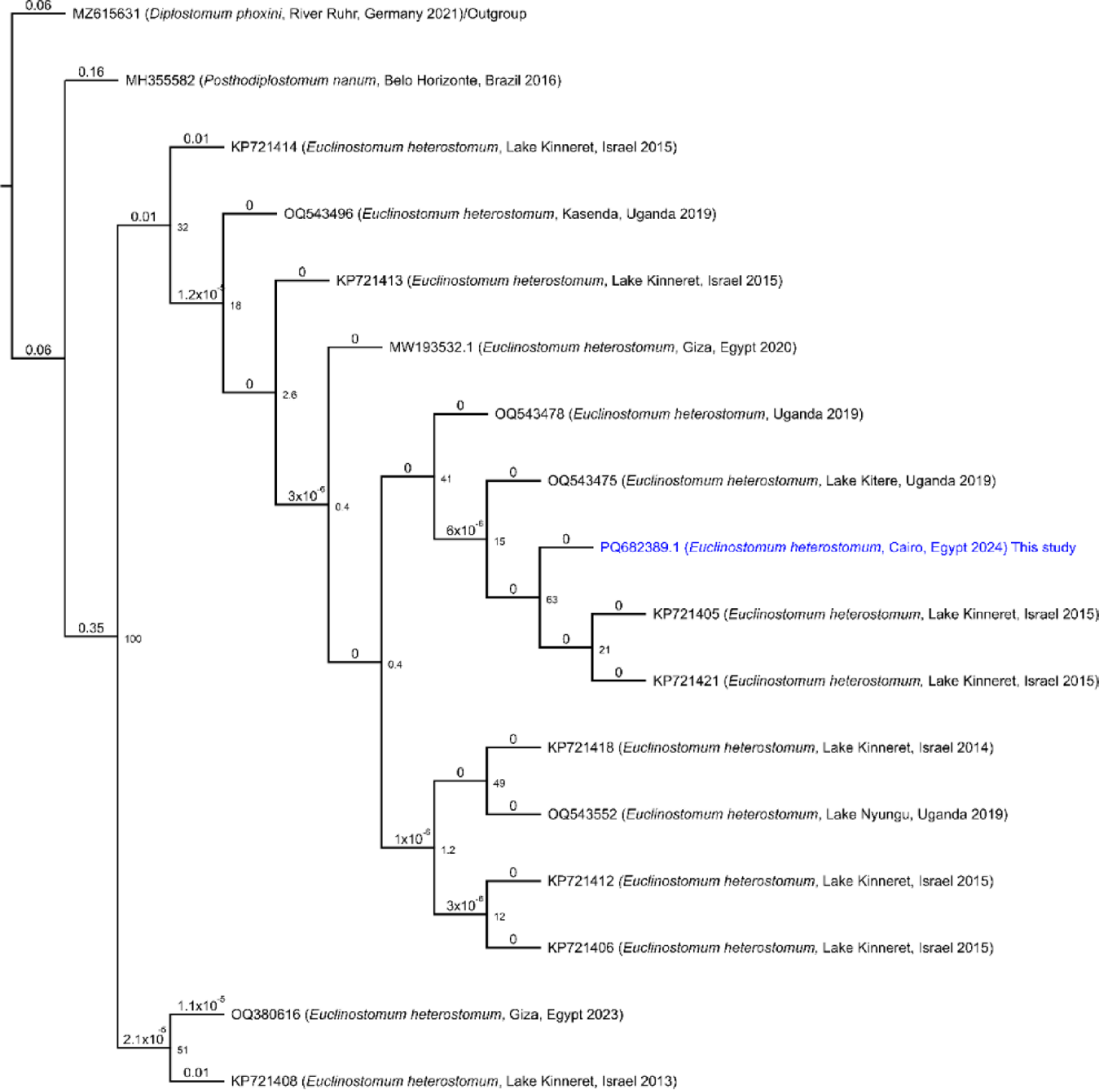
Phylogenetic Tree of *E. heterostomum* PQ682389.1 analyzed in the present study.

The phylogenetic analysis of *Clinostomum* sequences, including the newly identified *E. heterostomum* PQ682389.1, was conducted using the maximum likelihoodmethod (Fig. 2). The tree reveals that PQ682389.1 clusters closely with KP721421.1 and KP721415.1, indicating a strong evolutionary relationship. The genetic distance between PQ682389.1 and its nearest neighbor is 0.02 substitutions per site, suggesting moderate genetic similarity. Notably, several sequences exhibit a genetic distance of zero, signifying identical genetic profiles within specific clades.

#### Sequence homology and comparative analysis

A pairwise comparison of *E. heterostomum* PQ682389.1 against reference genomes demonstrated significant sequence homology (Supplementary Figs. 1 and 2). The comparison matrix indicates percent identity ranging from 98.51 to 100%, with the highest similarity observed between PQ682389.1 and KP721421.1, which exhibits a percent identity of 100%. The percent identity values highlight areas of high genetic similarity (closer to 100%) and greater genetic divergence (closer to 0%). Similarly, the patristic distance values effectively indicate high genetic similarity when closer to 0 and greater genetic divergence as they increase, underscoring the molecular diversity among the analyzed sequences.

This phylogenetic tree shows the relationships among various *E. heterostomum* sequences, with the sequence analyzed in this study (PQ682389.1) highlighted in blue. The tree is rooted using *Diplostomum phoxini* (accession MZ615631) as an outgroup to provide evolutionary context. The tree was constructed using the Maximum Likelihood method based on the Jukes-Cantor substitution model, with 1000 bootstrap replicates to assess branch support. Branch labels include GenBank accession numbers and genetic distances (substitutions per site) from the nearest sequence; branches with zero distance represent identical sequences. Sequences originate from diverse geographical locations, illustrating the genetic variation among *E. heterostomum* isolates.

### Phylogeny and sequence matrix for *C. complanatum* PQ876096.1

**Fig. 3:**
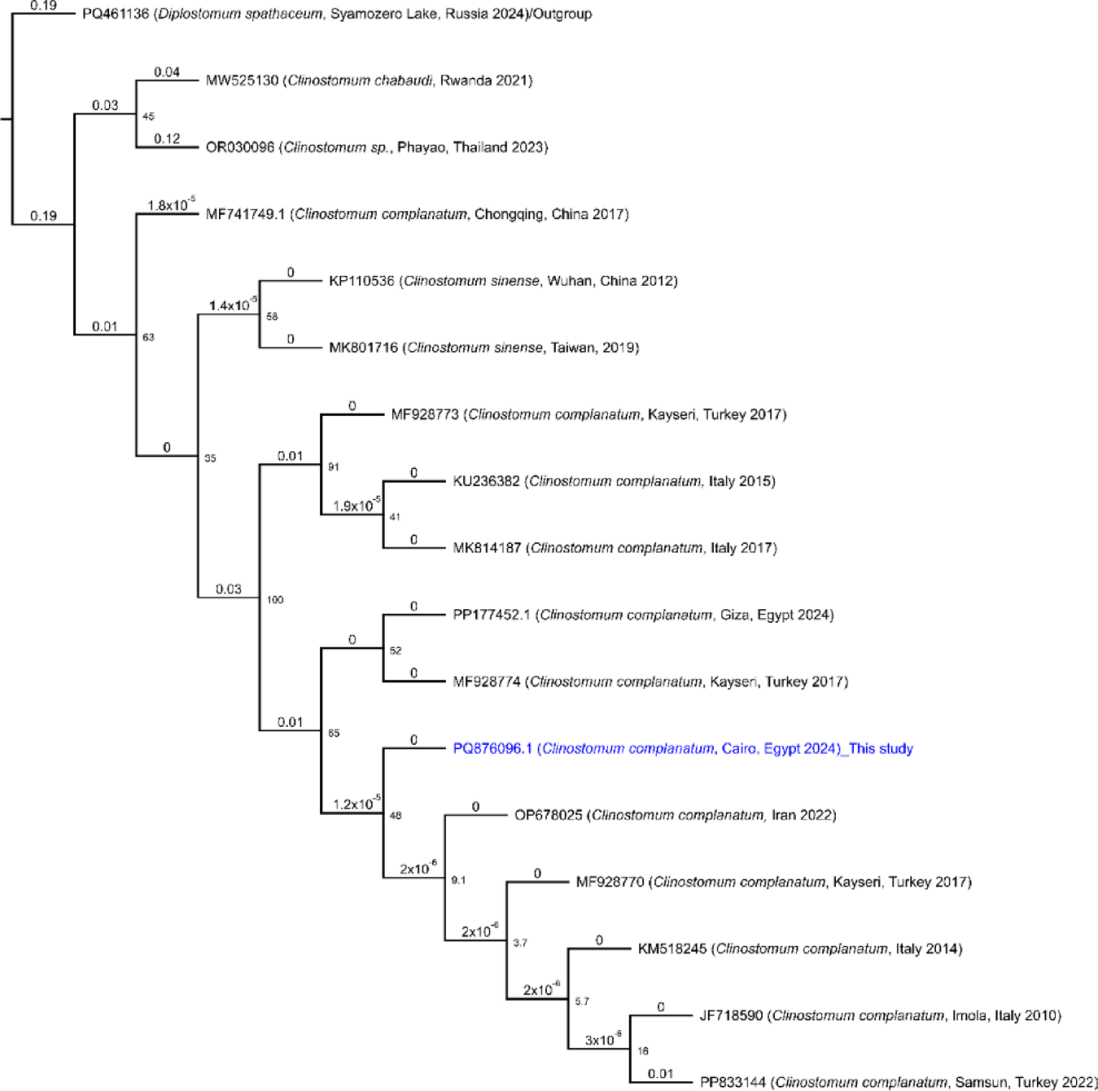
Phylogenetic tree illustrating genetic relationships among selected sequences with emphasis on PQ876096.1.

Further phylogenetic evaluation focused on the sequences *C. complanatum* PQ876096.1 revealed distinct genetic relationships (Fig. 3) and (Supplementary Figs. 3 and 4). The phylogenetic tree for PQ876096.1 illustrates its placement within a specific clade, closely related to OP678025.1 and MF928770.1 with a genetic distance of 0.00 substitutions per site. This positioning suggests PQ876096.1 belongs to a highly conserved lineage, with minimal genetic divergence from its closest relatives.

Similarly, the analysis of PQ876096.1 against multiple reference genomes highlights considerable sequence identity with PP833144.1 and PP177452.1 and variable sequence identity from MW525130.1 (91.53%) and OR030096.1 (85.97%) (Supplementary Figs. 3 and 4). The comparison matrix for PQ876096.1 underscores its unique genetic profile, contributing to the understanding of relevant biological or evolutionary insights. This matrix suggests a clear evolutionary divergence between closely related sequences (genetic distances of 0.00-0.01) and more distantly related sequences (genetic distances of 0.12–0.21), indicating distinct evolutionary lineages and possible adaptation events. This pattern indicates clusters of closely related sequences with high sequence conservation, separated from more divergent sequences that have accumulated more substitutions over evolutionary time.

This maximum likelihood phylogenetic tree depicts the genetic relationships among various *Clinostomum complanatum* sequences, including the sequence obtained in this study (highlighted in blue: PQ876096.1_This study). The tree is rooted using *Diplostomum spathaceum* (accession PQ461136) as an outgroup to establish evolutionary direction and infer common ancestry. The analysis was conducted with the Jukes-Cantor substitution model and 1,000 bootstrap replicates. Branch lengths represent genetic distances measured as substitutions per site; branches with zero length indicate identical or nearly identical sequences. Accession numbers and geographic origins are provided for all sequences, illustrating the genetic diversity and evolutionary relationships within *C. complanatum.*

### Oxidative stress of *C. complanatum* and *E. heterostomum* on infected fish

#### Biochemical markers of oxidative stress in *C. complanatum*

**Fig. 4:**
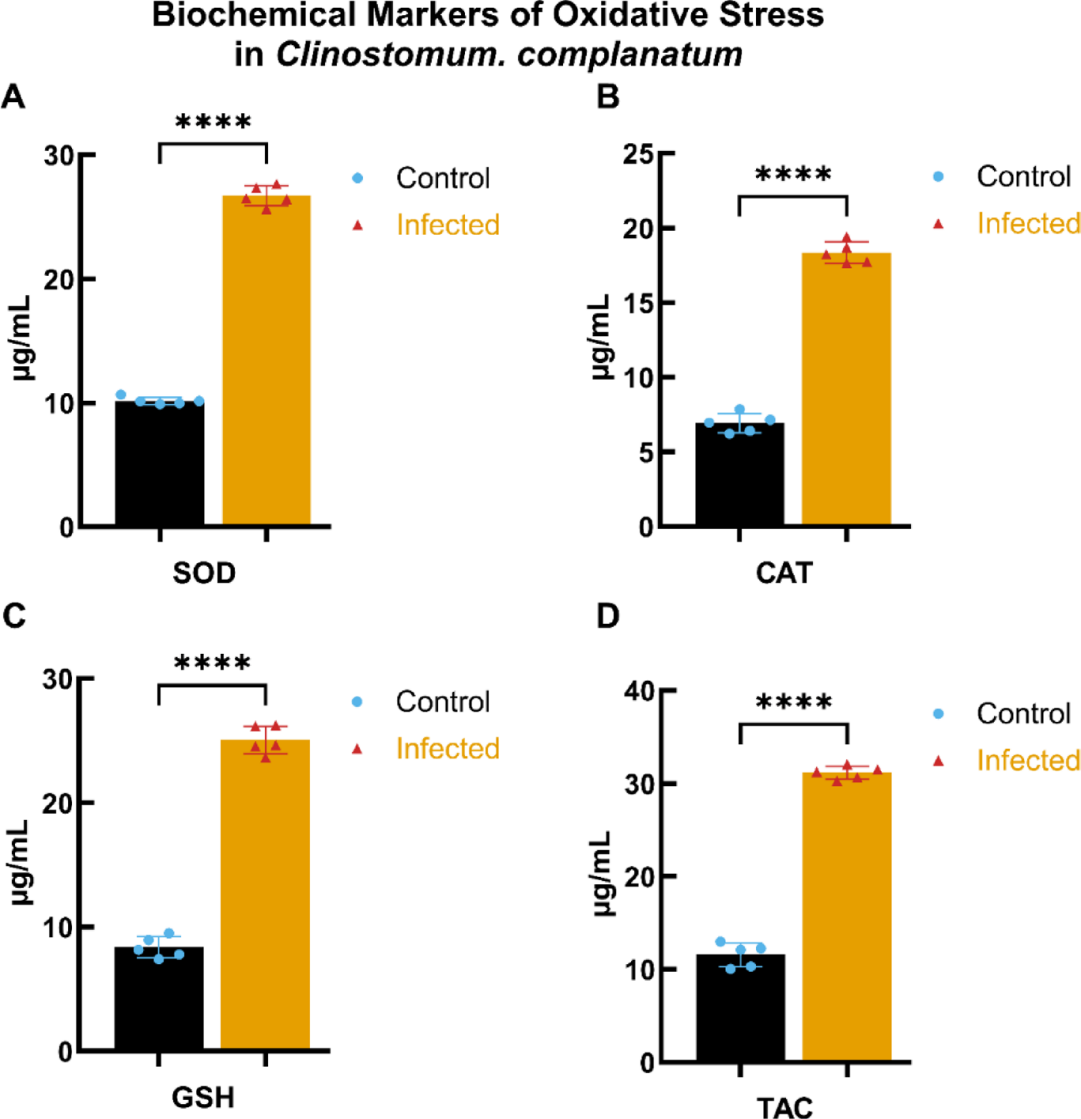
Biochemical markers of oxidative stress in *C. complanatum*. This figure presents the levels of various biochemical markers indicative of oxidative stress in *C. complanatum* under infected and control conditions. (**A**–**D**) display the concentrations (µg/mL) of Superoxide Dismutase (SOD) (**A**), Catalase (CAT) (**B**), Glutathione (GSH) (**C**), and Total Antioxidant Capacity (TAC) (**D**). Data are expressed as mean ± standard deviation (SD) for each group (*n* = 5), with statistical significance determined using a two-tailed Student’s t-test. Asterisks indicate significance levels, where ****p < 0.0001.

The assessment of oxidative stress markers in *C. complanatum* revealed significant alterations in biochemical parameters between infected and control fish. As illustrated in Fig. 4, the concentrations of superoxide dismutase (SOD) (Fig. 4A), Catalase (CAT) Fig. 4B), Glutathione (GSH) (Fig. 4C), and TAC (Fig. 4D) were markedly elevated in infected fish compared to controls. Specifically, SOD levels increased significantly (**** *p* < 0.0001), indicating an enhanced enzymatic response to oxidative damage. Similarly, CAT and GSH levels also exhibited significant increases (**** *p* < 0.0001), while TAC showed a pronounced elevation under infection conditions. These results suggest a robust antioxidant response to oxidative stress in*C. complanatum*-infected subjects.

#### Biochemical markers of oxidative stress in *E. heterostomum*

**Fig. 5:**
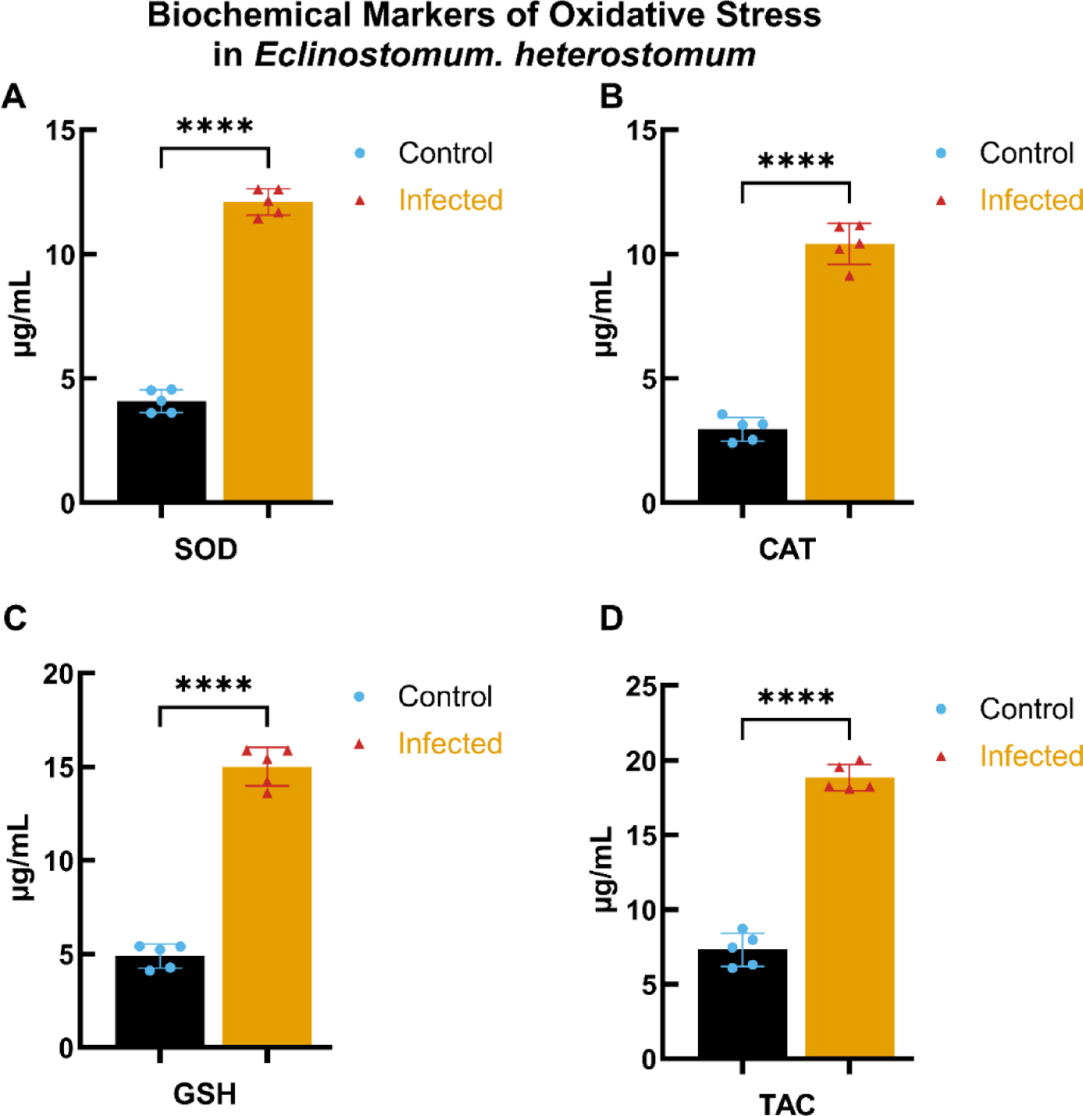
Biochemical markers of oxidative stress in *E. heterostomum*. This figure presents the levels of key biochemical markers of oxidative stress in *E. heterostomum* under control and infected conditions. (**A**–**D**) illustrate the concentrations (µg/mL) of superoxide dismutase (SOD), catalase (CAT), glutathione (GSH), and total antioxidant capacity (TAC), respectively. Data are expressed as mean ± standard deviation (SD), with each group comprising a sample size of *n* = 5. Statistical significance was determined using a two-tailed Student’s t-test, with a *p* < 0.0001 indicated by ****.

The oxidative stress profile for *E. heterostomum*is depicted in Fig. 5, showcasing SOD, CAT, GSH, and TAC levels under control and infected conditions. The data indicate significant increases in all measured biomarkers of oxidative stress in infected fish relative to controls (**** *p* < 0.0001). This trend is consistent with the findings for *C. complanatum*, underscoring a similar adaptive response to increased oxidative stress in both infections.

### Effect of *C. complanatum* and *E. heterostomum* infection on some cytokine’s parameters (gene expression analysis)

#### Immune response markers in *C. complanatum*-infected subjects

**Fig. 6:**
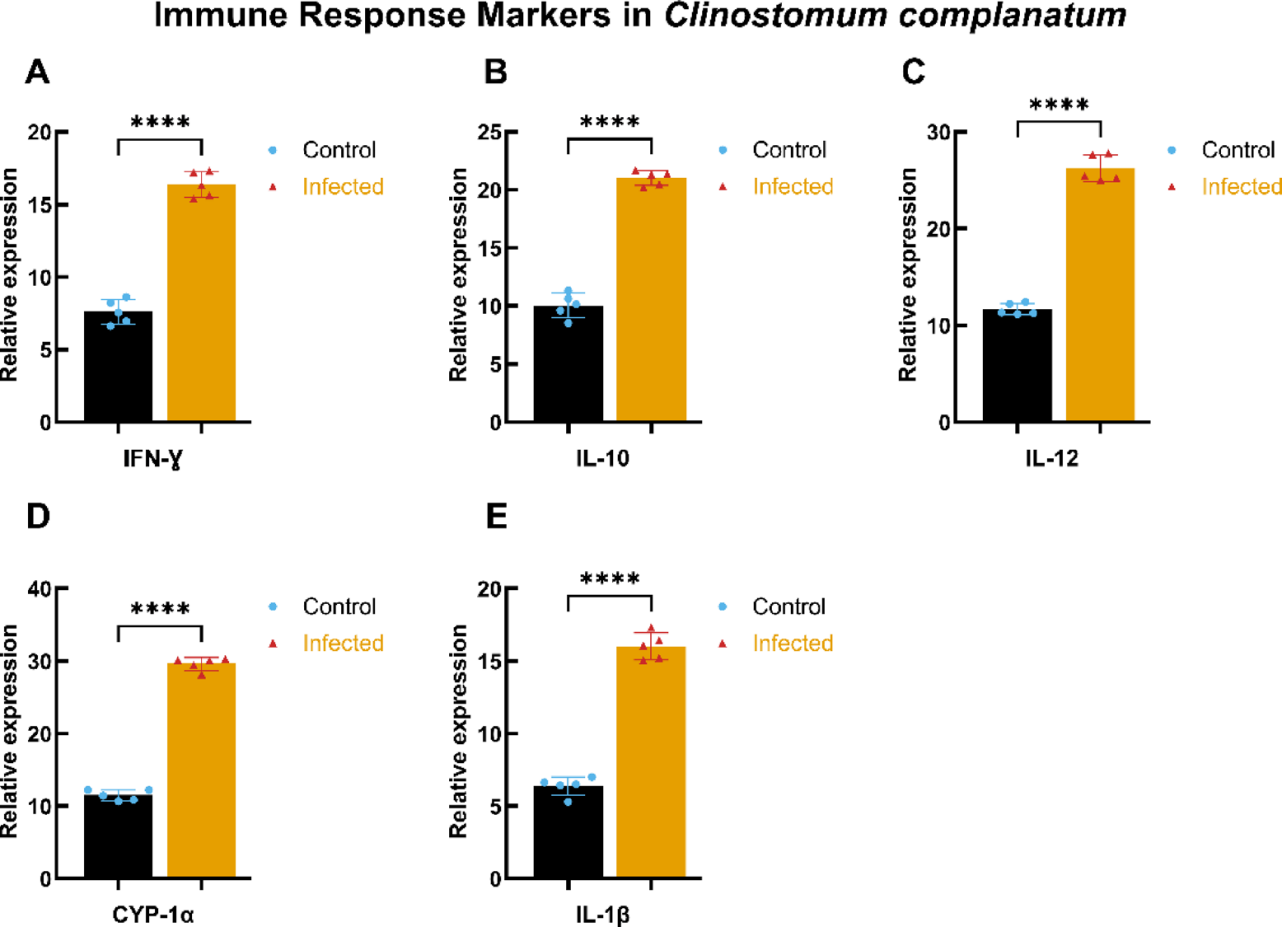
Immune response markers in *C. complanatum* infection. This figure presents the relative expression levels of key immune response markers from fish infected with *C. complanatum* compared to control subjects. (**A**–**E**) Illustrate the levels of *IFN-γ*,* IL-10*,* IL-12*,* CYP-1α*, and *IL-1β*, respectively. Data are expressed as mean ± standard deviation (SD) with statistical significance indicated by asterisks (****p < 0.0001).

The immune response was assessed through the measurement of key cytokines and immune markers. Figure 6 presents the concentrations of *IFN-γ* (Fig. 6A), *IL-10* (Fig. 6B), *IL-12* (Panel C), *CYP-1α* (Fig. 6D), and *IL-1β* (Fig. 6E) in infected versus control fish. All markers demonstrated significant elevations in the infected group (**** *p* < 0.0001). Notably, *IFN-γ* and *IL-12* levels were particularly high, suggesting a strong Th1-type immune response. In contrast, *IL-10* levels were also elevated, indicating a potential regulatory role in the immune response during infection.

#### Immune response markers in *E. heterostomum* infection

**Fig. 7:**
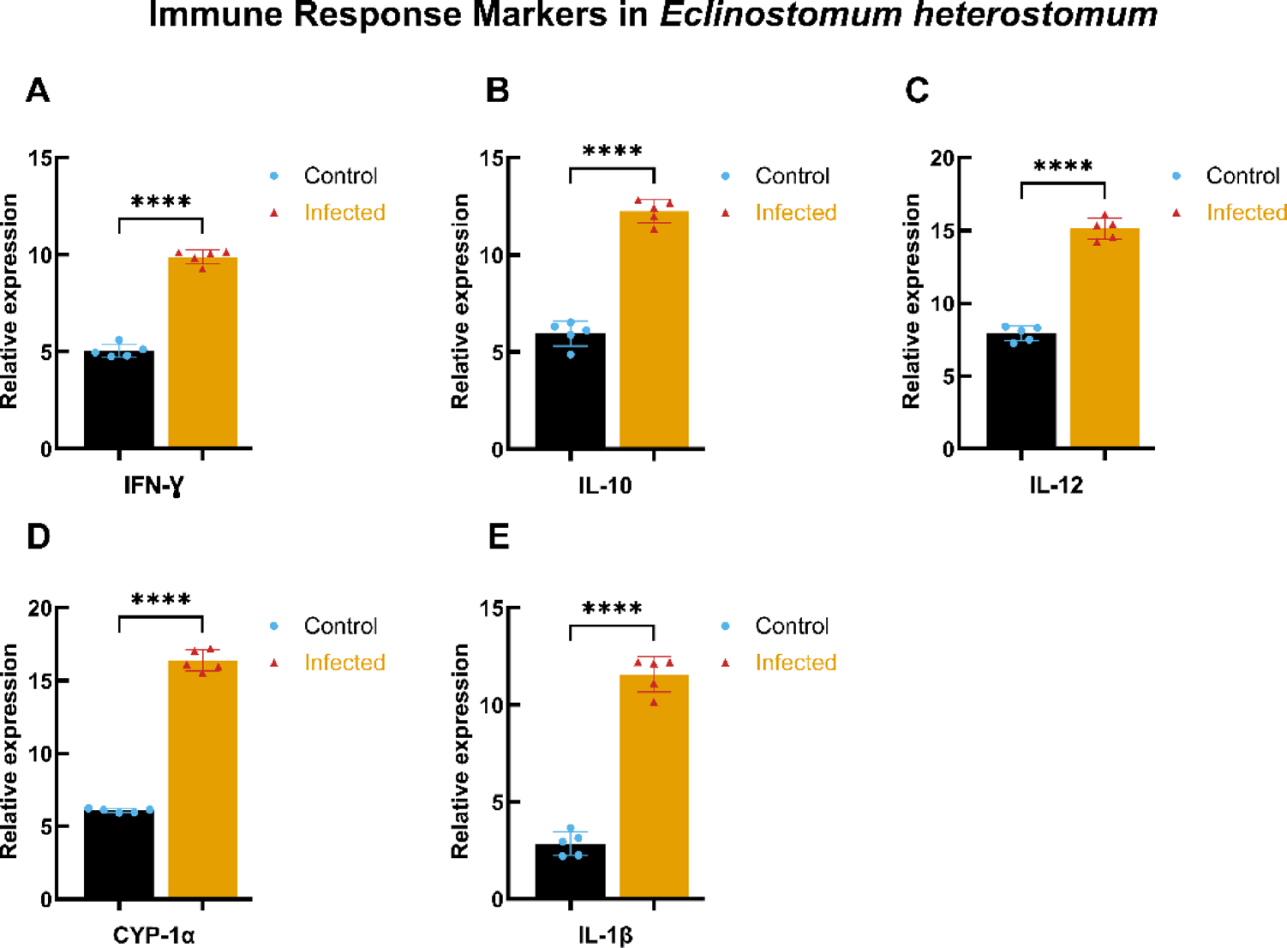
Immune response markers in *E. heterostomum* infection. This figure presents the relative expression levels of key immune response markers measured in control and infected groups of *E. heterostomum*. (**A**–**E**) depict levels of *IFN-γ*,* IL-10*,* IL-12*,* CYP-1α*, and *IL-1β* expressed in µg/mL. Data are represented as mean ± standard deviation (SD), with statistical significance assessed using a two-tailed Student’s t-test. Asterisks indicate significant differences, with **** denoting *p* < 0.0001 between the control (blue circles) and infected (red triangles) groups.

Similar trends were observed in the immune response markers for *E. heterostomum* infection as shown in Fig. 7. The levels of IFN-γ, IL-10, IL-12, CYP-1α, and IL-1β (Panels A-E) were significantly higher in infected fish compared to control subjects, with statistical significance denoted by **** *p* < 0.0001. These findings reinforce the observation of a vigorous immune response against *E. heterostomum*, characterized by elevated pro-inflammatory cytokines.

### Histopathological findings

**Fig. 8:**
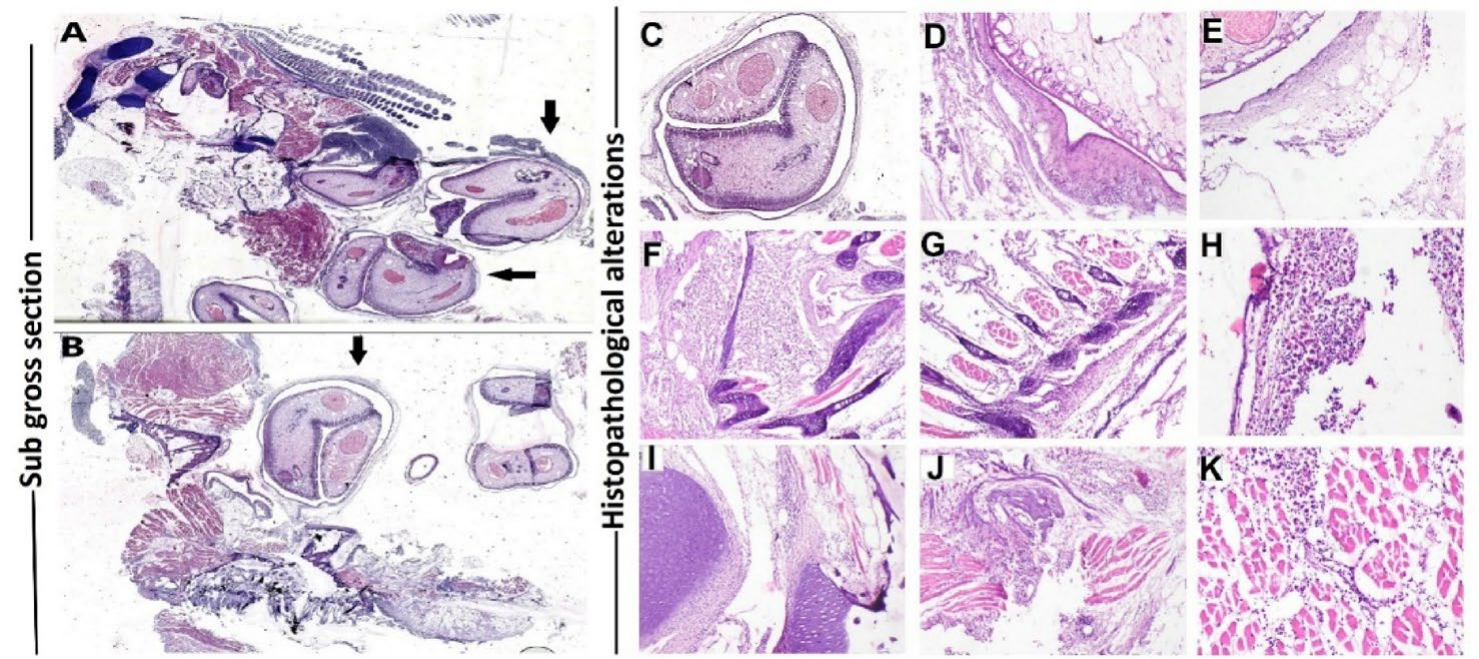
Left panel (**A**,**B**): Sub gross section from gills of infected fish showing multiple variable-sized encysted metacercaria cysts attached to the entire gill structures(arrows). (H and E stained section). Right panel: (**C**–**K**) Photomicrograph for gills of infected fish showing: encysted metacercaria cyst with dense cuticle, acetabulum (**C**-40x), the parasite enclosed by a dense fibrous collagenous capsule (**D**-100x), intense inflammatory cell infiltration in the fibrous capsule (**E**-100x), marked necrotic and inflammatory reaction surrounding the cartilaginous tissue (**F**-100x), severe destruction of gill lamellae with severe inflammatory reaction involving the gill lamellae and extending into entire gill structures (**G**-100x), intense aggregation of EGCs admixed with mononuclear cells in gill arch (**H**-200x), marked necrosis and inflammation of cartilaginous tissue in the vicinity of encysted metacercaria cyst (**I**-100x), interstitial edema with inflammatory cells infiltration mainly EGCs in the muscular tissue (**J**-100x), coagulative necrosis of myocytes with mononuclear cells infiltration in interstitial tissue (**K**-200x). (H and E stained histological section).

**Fig. 9:**
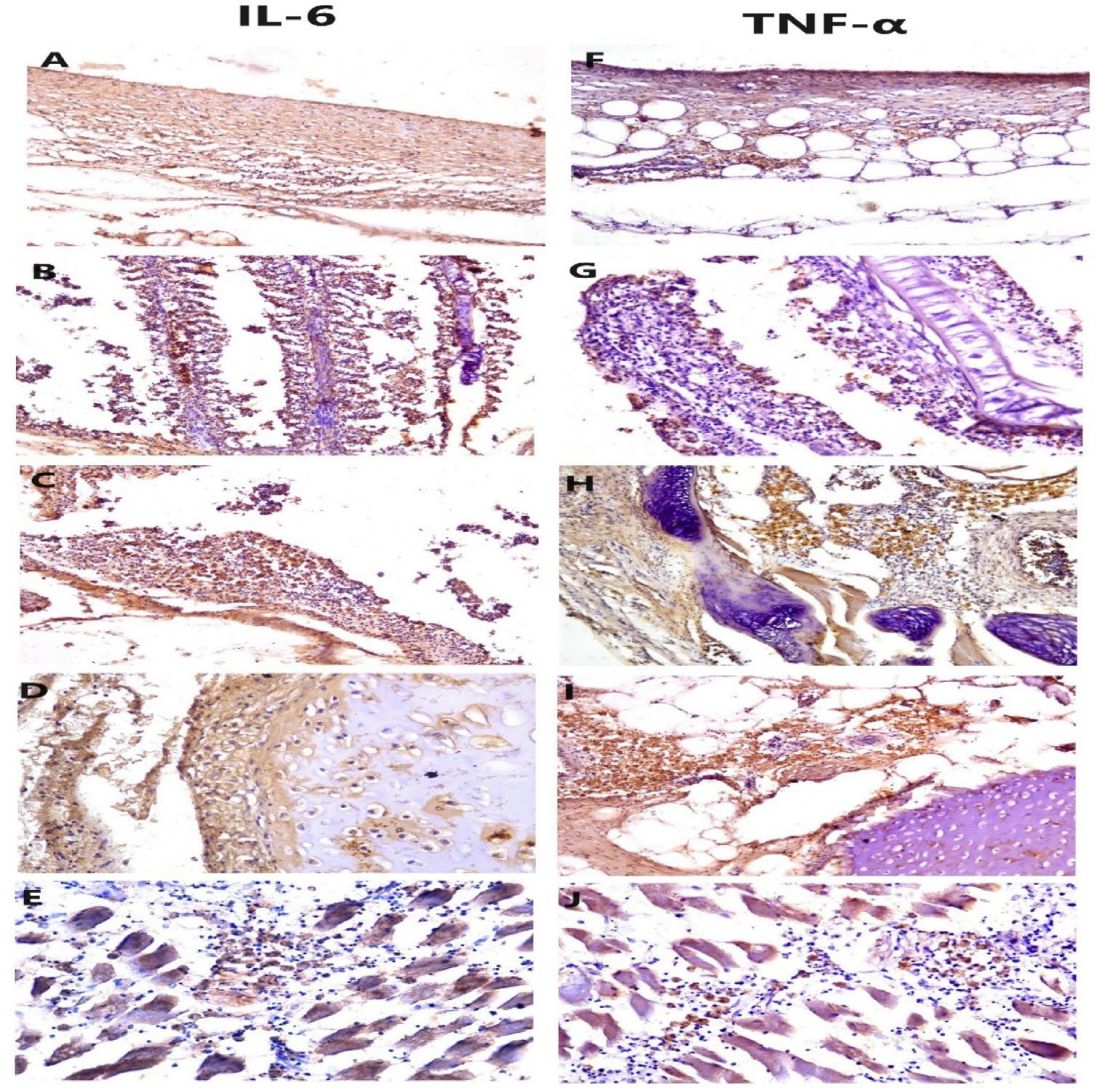
Immunohistochemistry (IHC) expression of IL-6 and TNF-α in tissues of infected fish, positive nuclear and cytoplasmic expression of IL-6 in the fibrous tissue of parasitic cysts and nuclear expression within the inflammatory cells (**A**-200x), inflammatory cells infiltrating the gill lamellae (**B**-200x), EGCs (**C**-200x), chondrocytes (**D**-200x), andthe inflammatorycells infiltrating the muscle interstitial tissue (**E**-200x). Positive nuclear and cytoplasmic expression of TNF-α in the parasitic fibrous wall and adipose tissue (**F**-200x), inflammatory cells infiltrating the gill lamellae (**G**-200x), EGCs surroundingthe cartilaginous tissue (**H**-200x), EGCs infiltrating the interstitial tissue (**I**-200x) and inflammatory cells in muscle interstitialtissue (**J**-200x).

The gill structures revealed multiple parasitic cysts containing encysted metacercaria of *C. complanatum* with a dense collagenous fibrous capsule firmly attached to gill structures (Fig. 8, left panel). The structure of EMC is characterized by a dense eosinophilic cuticle, acetabulum, and intestinal ceca (Fig. 9). The thick capsule surrounding the parasite was incorporated into the host connective tissues; there was an intense inflammatory cell infiltration into the capsular wall. The gill arch showed extensive necrosis of cartilaginous and muscular tissue comprising the gill structures with destruction of the lamellar epithelium of gill lamellae, the gill arch showed extensive eosinophilic granular cells (EGCs) admixed with mononuclear cells, the muscles showed coagulative necrosis, interstitial edema, inflammatory cells infiltration, and the necrotic and inflammatory reaction extending into adjacent cartilage (Fig. 8, Right panel).

The immunohistochemical characterization of IL-6 and TNF-αexpressions in the infected tissue was evident in fibrous tissue, lamellar epithelium of gills, mononuclear cells, and EGCs, the expression was cytoplasmic and nuclear in the target cells (Fig. 9).

**Fig. 10:**
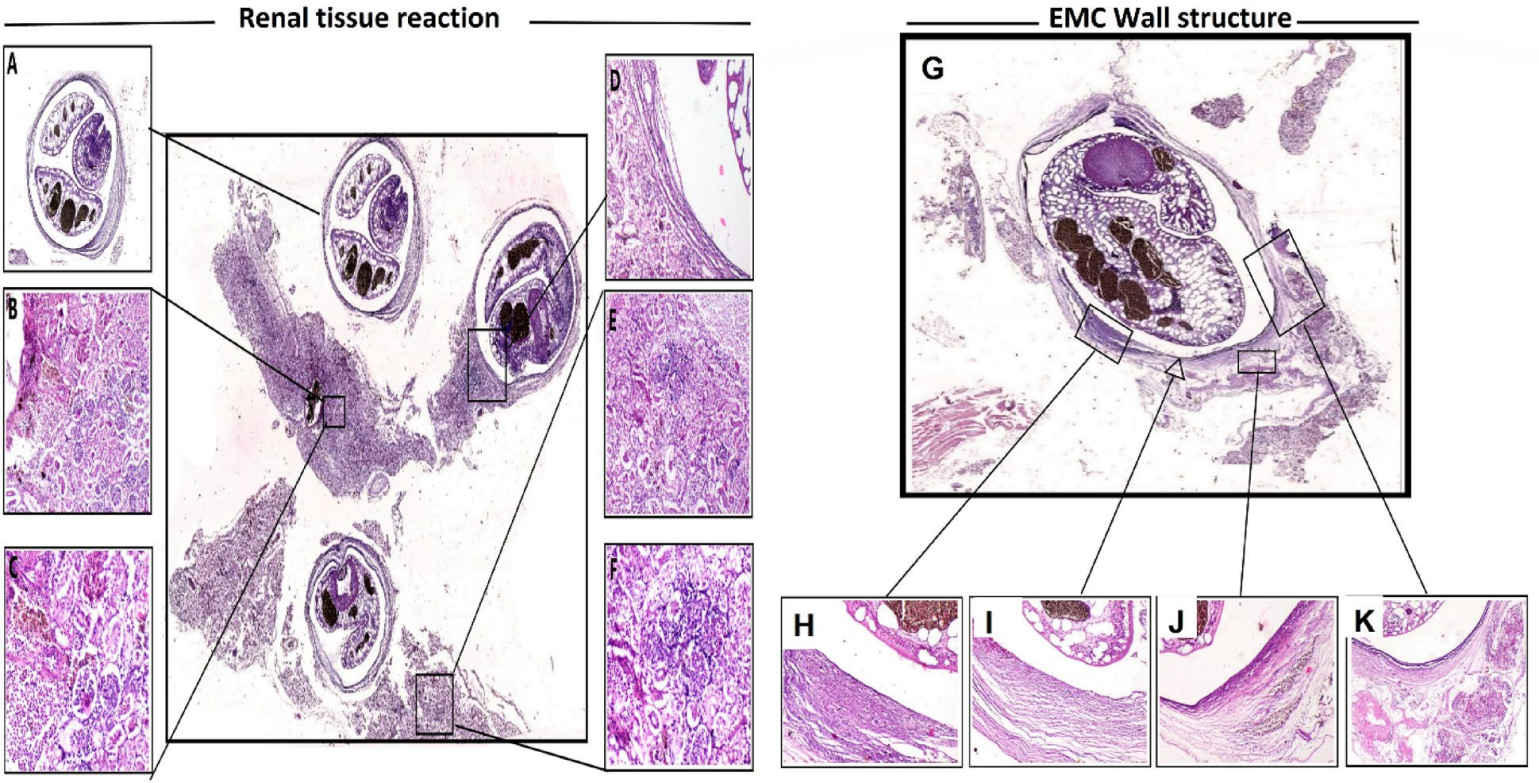
Left Panel Subgross photomicrograph showing multiple encysted metacercariae of *E. heterostomum* with renal tissue reaction surrounding the cysts, the parasite enclosed by dense fibrous connective tissue capsule (**A**-40x), severe congestion of blood vessels in renal interstitial tissue (**B**-100x), activation of melanomacrophage center with periglomerular aggregation of melanomacrophages admixed with mononuclear cells (**C**-200x), the dense fibrous capsule surrounding the metacercaria incorporated into host renal interstitial tissue (**D**-100x), marked inflammatory reaction extending diffusely in renal interstitial tissue (**E**-100x), severe necrosis of renal tubular epithelium and interstitial congestion and hemorrhages (**F**-200x). (H and E-stained histological section). Right Panel: Subgross photomicrograph of encysted metacercariae of *E. heterostomum* the enclosed parasite structures consisted of the cuticle, acetabulum, intestinal ceca (**G**-40x), the cyst wall consisted of dense collageneous tissue (**H**-100x), focal hemorrhage in the wall of fibrous capsule (**I**-100x), the parasitic capsule infiltrated by inflammatory cells with melanomacrophages (**J**-100x), renal interstitial hemorrhage in the area surrounding the encysted metacercaria (**K**-100x). H and E-stained histological section).

The sub-gross examination of the kidney of infected fish showed the presence of multiple large encysted metacercaria cysts of *E. heterostomum* attached with dense thick fibrous tissue that incorporated into host renal tissue (Fig. 10, left panel).

The histopathological alterations in renal tissue included extensive tubular vacuolization and necrosis. The renal intersititum exhibited congestion of peritubular blood vasculatures, interstitial hemorrhage. In addition, the inflammatory reaction was evident in renal tissue that extended into the parasitic cyst wall, the inflammatory reaction characterized by mononuclear cell infiltration with melano-macrophages (Fig. 10, left panel). The parasitic cyst consisted of dense collagenous fibers that were incorporated into the host renal tissue and enclosed the entire structures of *E. heterostomum* metacercariae (Fig. 10, Right panel).

**Fig. 11:**
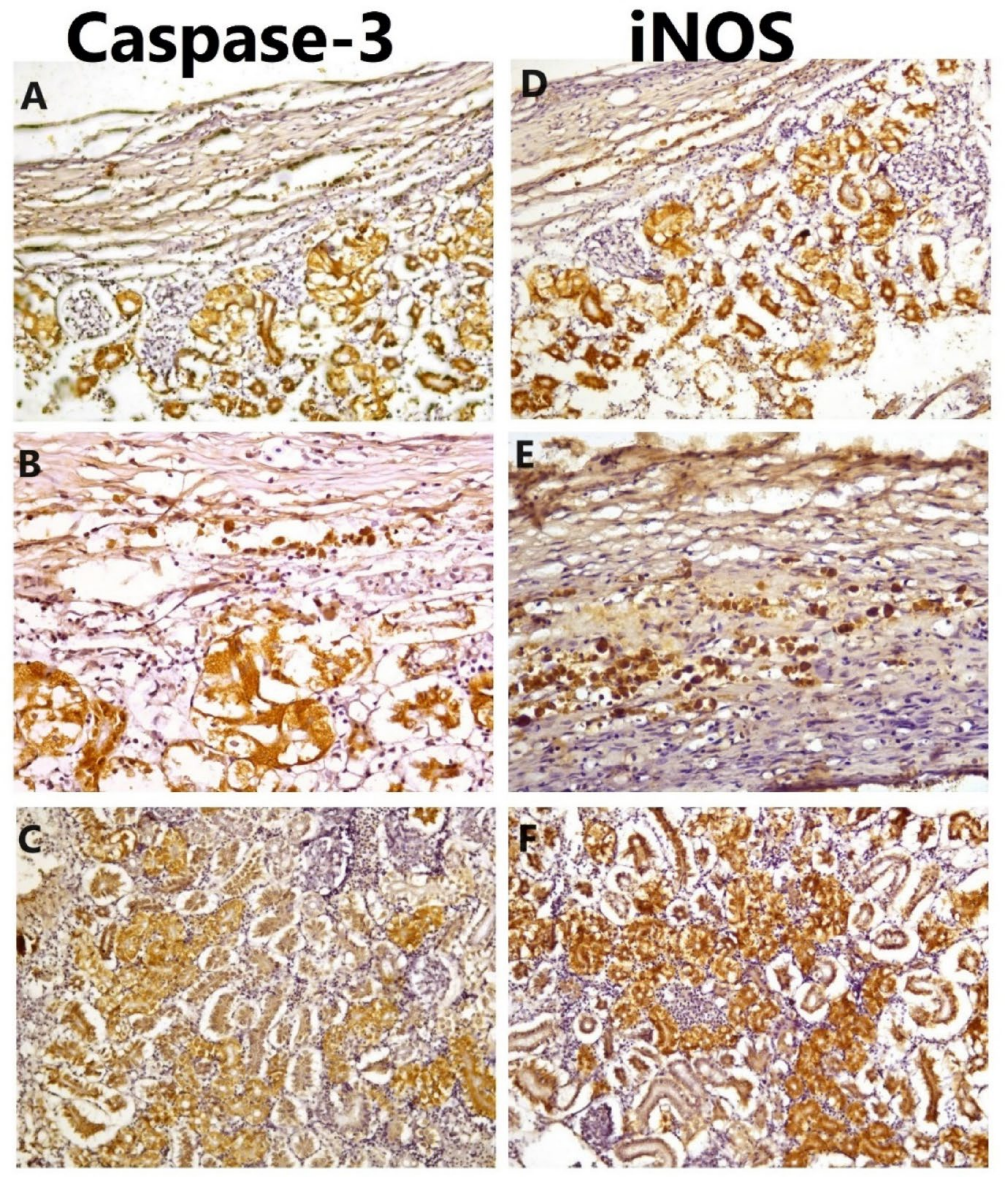
Immunohistochemistry (IHC) expression of caspase-3 and iNOSin renal tissue of infected fish, positive expression of caspase-3 in tubular epithelium directly surrounding the parasitic cyst (**A**-100x), the expression was nuclear and cytoplasmic in renal tubular epithelial cells (**B**-200x), the expression extending deeply into the tubular epithelium (**C**-100x). The positive reaction of iNOSwas evident in renal tissue in the vicinity of the parasitic cyst (**D**-100x), and positive expression was evident in EGCs (**E**-x200) and extending deeply into renal tubular epithelium (**F**-100x).

The immunohistochemical expression of caspase-3 was evident in tubular epithelium that was nuclear and cytoplasmic, while the iNOSexpression was evident in tubular epithelium and mononuclear cells; the expressions of both markers were evident in renal tissue adjacent to the parasitic capsule and extending into renal parenchyma (Fig. 11).

## Discussion

Parasitic diseases are often transmitted via fish, and species within the Clinostomidae family are widespread across the globe, primarily through piscivorous birds. Juvenile worms can significantly harm fish, leading to deformities that reduce their commercial and ornamental value^29^. In the present study, molecular identification of *C. complanatum* and *E. heterostomum* was successfully achieved through COXI gene sequencing, confirming the species and clarifying their phylogenetic relationships. This approach is consistent with previous studies, such as Moszczynska et al.^13^who identified the COXI gene as a dependable molecular marker for species differentiation in trematodes. Similarly, Mahdy et al. 2022 and 2024^30,31^ demonstrated that COXI-based analyses are particularly useful in distinguishing morphologically similar species within the *Clinostomum* and *Euclinostomum* genera.

Phylogenetic analysis of *C. complanatum* sequences revealed that the isolates clustered closely with those previously reported from Turkey^32^ and Africa^33^suggesting a notable degree of genetic conservation across geographically distinct regions. Most sequence identities exceeded 90%, which is typically considered robust, as it indicates a high level of genetic similarity. These findings support the notion of genetic consistency among *C. complanatum* isolates. Similarly, Younis et al.^17^ reported genetic homogeneity among isolates from different fish hosts.

For *E. heterostomum*, phylogenetic analysis confirmed its close genetic relationship with isolates from Egypt, Italy, Uganda, Belgium, and other countries. The sequence PQ682389.1 from the present study clustered with those from Purivirojkul et al. and Mahdy et al.^34,35^suggesting a stable genetic profile within *E. heterostomum*. Comparative sequence analysis revealed minimal genetic divergence, confirming the genetic stability of *E. heterostomum* across various ecological conditions, as reported by Caffara et al.^33^. Li et al.^36^ observed similar intraspecific variability in *Clinostomum* species from aquaculture environments, underscoring the influence of environmental pressures on genetic diversity. The use of COXI sequencing in the present study, alongside morphological identification, helped overcome the limitations of relying solely on morphology for distinguishing between closely related genera, as emphasized by Dzikowski et al. and Mahdy et al.^37,38^.

The present study assessed the expression of several immune genes in the gills, and kidneys of infected and non-infected fish. Fish frequently harbor parasites, yet mucus released from these regions serves as a barrier, helping to reduce parasite load. According to Dickerson and Findly^39^parasitic protozoan infections can stimulate an immunological response in *O. niloticus* with infection intensity ranged from 2 to 12 cysts per fish averaging 5 cysts, leading to upregulated gene expression.

Inflammatory cytokines are typically activated in response to parasite-induced injury^1^. Cytokines are essential mediators of the immune response to infectious parasites. Recent research has utilized cytokine levels to detect immune responses in fish infected with parasites. Younis et al. and Barrett et al.^17,40^ studied the immune mechanisms of TNF-α and IL-1β, which help control infections in rainbow trout and Nile tilapia, respectively. Additionally, TNF-α, secreted by macrophages as reported by Tu et al.^41^plays a key role in the immune defense. In fish, IL-6 and IL-10 play crucial roles in both innate and adaptive immune responses. IL-6 is generally considered a pro-inflammatory cytokine, while IL-10 is an anti-inflammatory cytokine. They work together to regulate the immune response, with IL-6 often promoting inflammation and pathogen clearance, and IL-10 dampening the immune response to prevent excessive tissue damage. In the present study, the expression of cytokines such as IFN-γ, IL-10, IL-12, CYP-1α, and IL-1β significantly increased in fish infected with *Clinostomum* and *Euclinostomum*, suggesting an immune response aimed at combating the parasitic infection. These cytokines are involved in various biological processes, including inflammation, fibrosis, cell growth, and immune response^42^.

The present study found significant changes in oxidative stress markers in fish infected with clinostomid parasites, indicating an increased production of reactive oxygen species (ROS) and reactive nitrogen species (RNS) by the host in response to infection. Superoxide dismutase (SOD) converts superoxide radicals into hydrogen peroxide, which can cause cellular damage if not efficiently broken down by catalase (CAT)^43,44^. Infected fish exhibited elevated levels of SOD, CAT, glutathione (GSH), and total antioxidant capacity (TAC) compared to healthy counterparts, reflecting the oxidative stress burden and the compensatory antioxidant response. Oxidative stress arises when the generation of ROS exceeds the body’s ability to neutralize them, leading to damage of essential biomolecules. According to Esmaeilnejad et al. and Abbaci et al.^45,46^ROS are generated to combat infections; however, their excessive accumulation can result in oxidative stress. While ROS help defend against pathogens, they may also damage host cells by inducing lipid peroxidation, compromising membrane permeability, and leading to hemoglobin release and membrane damage^42^. Likewise, oxidative damage to DNA and proteins may result in mutations and loss of metabolic functions^9^. Antioxidants, such as GSH and SOD, are therefore crucial in managing oxidative stress and maintaining cellular homeostasis^47^. The consistent upregulation of both immune-related cytokines and oxidative stress markers in infected fish indicates a coordinated physiological response to clinostomid infection. Specifically, the simultaneous elevation of pro-inflammatory cytokines (e.g., IFN-γ, IL-1β) and antioxidant enzymes (e.g., SOD, CAT, GSH, TAC) suggests that the host’s immune activation is tightly linked to redox imbalance. This connection reinforces the interpretation that inflammation and oxidative stress act in concert as part of the host defense mechanism.

Histopathological analysis of infected fish gills and kidneys revealed severe changes, primarily due to parasitic infections by *C. complanatum* and *E. heterostomum*. Significant damage to gill structures, with cyst walls integrated into the body, was observed, supporting the gills as a primary target for *C. complanatum*^10,48,49^. The study also showed an intense inflammatory response in gill structures, marked by increased expression of pro-inflammatory cytokines like IL-6 and TNF-α. This cytokine upregulation correlated with necrotic and inflammatory reactions, indicating that inflammatory cell infiltration was linked to cytokine production and tissue damage. Similar findings in zebrafish and turbot infected with Mycobacterium marinum and *Enteromyxum scophthalmi* also highlighted the role of TNF-α in tissue damage^50–52^. Bari et al.^53^ described the histopathological alterations induced in liver tissue by *Euchinostoma heterostomum* in *Channa punctata*, revealing marked inflammatory and degenerative changes in the liver of infected fish as a result of direct parasitic encystation.

In addition to gill pathology, kidney infections with *E. heterostomum* also led to severe damage, confirming the kidney as a major site for encystation. Previous research identified multiple organs, including the liver, kidney, eyes, skin, and coelomic cavity, as sites for *E. heterostomum* encystment^35^. The inflammatory and necrobiotic reactions observed in the renal tissues were directly associated with encysted *E. heterostomum* metacercariae. The upregulation of caspase-3 and iNOS expression in renal tissues was linked to increased inflammatory responses and oxidative stress, which activated apoptotic pathways, as observed in other studies on oxidative stress-induced tissue damage^5,54^.

## Conclusion

This study provides comprehensive evidence on the impact of *C. complanatum* and *E. heterostomum* infections in Nile tilapia. Molecular identification using COXI gene sequencing confirmed both parasite species and their close genetic links to isolates from other regions, indicating widespread distribution. Infected fish showed significant upregulation of immune genes (IFN-γ, IL-10, IL-12, IL-1β, and CYP-1α) and elevated oxidative stress markers (SOD, CAT, GSH, and TAC), reflecting strong immune activation and physiological stress. Severe tissue damage, including necrosis, fibrosis, and inflammatory infiltration, was confirmed in gills and kidneys, with localized expression of inflammatory markers. These findings underline the serious health risks posed by clinostomid infections to tilapia and aquaculture productivity. The integrated molecular, immunological, and pathological data provide essential insights for disease monitoring and control programs. Future research should explore ecological factors influencing parasite spread, assess long-term health impacts on fish, and develop targeted prevention or treatment strategies to safeguard aquaculture systems.

## Supplementary Information

Below is the link to the electronic supplementary material.

Supplementary Material 1

## Data Availability

All the authors declare that all the data supporting the results reported in our article were included in this article only. The datasets generated or analyzed during the current study are available in the GENEBANK repository [PQ682389, PQ876096].
