# Supplementary material for: Molecular and pathological insights into gene expression and oxidative stress in Clinostomum complanatum and Euclinostomum heterostomum
Source: Sci Rep. 2025 Oct 28;15:37586. doi: 10.1038/s41598-025-16469-5 (PMC12569213; doi:10.1038/s41598-025-16469-5)
Supplement: Supplementary file 1 — Supplementary Material 1 [file 41598_2025_16469_MOESM1_ESM.docx]

**Supplementary Information**

**For**

**Molecular and Pathological Insights of *Clinostomum complanatum* and *Euclinostomum heterostomum*: Gene Expression, Oxidative Stress, and Immune Responses**

**Mai A. Salem^1^, Olfat A. Mahdy^1^, Mohamed A. El-Saied^2^, Mohamed S. Kamel^3^, Faten F. Mohammed^2,4^ and Reem M. Ramadan^1*^**

^1^Department of Parasitology Department, Faculty of Veterinary Medicine, Cairo University, Giza, Egypt.

^2^Department of Pathology, Faculty of Veterinary Medicine, Cairo University, Giza, 12211, Egypt.

^3^Department of Medicine and Infectious Diseases, Faculty of Veterinary Medicine, Cairo University, 12211, Giza, Egypt.

^4^Department of Pathology, College of Veterinary Medicine, King Faisal University, Al-Ahsa, 31982, Saudi Arabia.

***Corresponding author**: **Reem M. Ramadan**

**E-mail address:** [reem.montaser@cu.edu.eg](mailto:reem.montaser@cu.edu.eg)

**Supplementary Figures**

**Supplementary Fig. 1:** Comparison of Sequence Identity Percentages of *Euclinostomum heterostomum* PQ682389.1 against other Genomic Sequences. This figure presents the pairwise comparison matrix results of sequence homology from PQ682389.1 (this study) and other reference genomes identified by their accession numbers.

**Supplementary Fig. 2:** Comparison of Sequence Distance of *Euclinostomum heterostomum* PQ682389.1 against other Genomic Sequences. This figure presents the pairwise comparison matrix results of sequence homology from PQ682389.1 (this study) and other reference genomes identified by their accession numbers.

**Supplementary Fig. 3:** Sequence Comparison Identity Among Multiple Genomes Including the *Clinostomum complanatum* Sequence PQ876096.1. This figure presents the pairwise comparison matrix results of sequence homology from PQ876096.1 (this study) and other reference genomes identified by their accession numbers (e.g., QP676805.1, MFPI8270.1, etc.).

**Supplementary Fig. 4:** Sequence Comparison Distance Among Multiple Genomes Including the *Clinostomum complanatum* Sequence PQ876096.1. This figure presents the pairwise comparison matrix results of sequence homology from PQ876096.1 (this study) and other reference genomes identified by their accession numbers (e.g., QP676805.1, MFPI8270.1, etc.).

**Supplementary Table 1:** Accession Numbers, Species, and Countries of *Clinostomum* Species Included in This Study and Our Sequence.

| Accession number | Species | Country |
| --- | --- | --- |
| PQ876096.1 | Clinostomum complanatum isolate Fish cytochrome c oxidase subunit I (COX1) gene, partial cds; mitochondrial. | Egypt (This study) |
| PP833144.1 | Clinostomum complanatum voucher OMUPAR.874.23.01 cytochrome c oxidase subunit I (COX1) gene, partial cds; mitochondrial. | Turkey |
| PP177452.1 | Clinostomum complanatum isolate Comtil024 cytochrome c oxidase subunit I (COX1) gene, partial cds; mitochondrial | Egypt |
| OP678025.1 | Clinostomum complanatum voucher C11 cytochrome c oxidase subunit I (COX1) gene, | Belgium |
| MF928770.1 | Clinostomum complanatum isolate ERU-C.comp(2c-1) cytochrome oxidase subunit 1 (COI) gene, partial cds; mitochondrial. | Turkey |
| MW525130.1 | Clinostomum chabaudi isolate HV1819 cytochrome c oxidase subunit I (COX1) gene, partial cds; mitochondrial | Germany |
| OR030096.1 | Clinostomum sp. isolate PYO006.1 cytochrome c oxidase subunit I (COX1) gene, partial cds; mitochondrial. | Thailand |
| MF741749.1 | Clinostomum complanatum isolate C21 cytochrome oxidase subunit 1 (COI) gene, partial cds; mitochondrial | China |
| KP110536.1 | Clinostomum sinense voucher C.Op.Ca.Lzi.3 cytochrome oxidas subunit 1 (COI) gene, partial cds; mitochondrial. | Canada |
| MK801716.1 | Clinostomum sinensis isolate 102/18I1 cytochrome oxidase subunit I(COI) gene, partial cds; mitochondrial. | Italy |
| MF928773.1 | Clinostomum complanatum isolate ERU-C.comp(MetZ2) cytochrome oxidase subunit 1 (COI) gene, partial cds; mitochondrial | Turkey |
| MK814187.1 | Clinostomum complanatum mitochondrion, complete genome | USA |
| KU236382.1 | Clinostomum complanatum isolate 40/15 cytochrome oxidase subunit I (COI) gene, partial cds; mitochondrial | Italy |
| MF928774.1 | clinostomum complanatum isolate ERU-C. comp(MetZ3) cytochrome oxidase subunit 1 (COI) gene, partial cds; mitochondrial. | Turkey |
| JF718590.1 | Clinostomum complanatum voucher C.Sc. ITA3.5 cytochrome oxidase subunit 1 (COI) gene, partial cds; mitochondrial. | Italy |
| KM518245.1 | Clinostomum complanatum isolate 46/12_7 cytochrome oxidase subunit I (COI) gene, partial cds; mitochondrial | Italy |

**Supplementary Table 2:** Accession Numbers, Species, and Countries of *Euclinostomum heterostomum*Included in This Study and Our Sequence

| Accession number | Species | Country |
| --- | --- | --- |
| PQ682389.1 | Euclinostomum heterostomum isolate Fish cytochrome c oxidase subunit I (COX1) gene, partial cds; mitochondria | Egypt (This study) |
| KP721405.1 | Euclinostomum heterostomum isolate 349/13_3 cytochrome oxidase subunit I (COI) gene, partial cds; mitochondrial | Italy |
| KP721421.1 | Euclinostomum heterostomum isolate 55/14_12 cytochrome oxidase subunit I (COI) gene, partial cds; mitochondrial | Italy |
| OQ543475.1 | Euclinostomum heterostomum isolate 2019_UG_KAS11_BUL121_infection cytochrome c oxidase subunit I (COX1) gene, partial cds; mitochondrial | Belgium |
| OQ543478.1 | Euclinostomum heterostomum isolate 2019_UG_KAS11_BUL89_infection cytochrome c oxidase subunit I (COX1) gene, partial cds; mitochondrial | Uganda |
| KP721412.1 | Euclinostomum heterostomum isolate 55/14_2 cytochrome oxidase subunit I (COI) gene, partial cds; mitochondrial | Italy |
| KP721413.1 | Euclinostomum heterostomum isolate 55/14_3 cytochrome oxidase subunit I (COI) gene, partial cds; mitochondrial | Italy |
| KP721406.1 | Euclinostomum heterostomum isolate 349/13_4 cytochrome oxidase subunit I (COI) gene, partial cds; mitochondrial | Italy |
| OQ543496.1 | Euclinostomum heterostomum isolate 2019_UG_KAS13_BUL298_infection cytochrome c oxidase subunit I (COX1) gene, partial cds; mitochondrial | Uganda |
| KP721418.1 | Euclinostomum heterostomum isolate 55/14_9 cytochrome oxidase subunit I (COI) gene, partial cds; mitochondrial | Italy |
| OQ543552.1 | Euclinostomum heterostomum isolate 2019_UG_QE1_BUL54_infection cytochrome c oxidase subunit I (COX1) gene, partial cds; mitochondrial | Belgium |
| MW193532.1 | Euclinostomum heterostomum isolate Orechromus niloticus fish cytochrome c oxidase subunit I (COX1) gene, partial cds; mitochondrial | Egypt |
| KP721414.1 | Euclinostomum heterostomum isolate 55/14_4 cytochrome oxidase subunit I (COI) gene, partial cds; mitochondrial | Italy |
| OQ380616.1 | Euclinostomum heterostomum isolate MT13 cytochrome c oxidase subunit I (COX1) gene, partial cds; mitochondrial | Egypt |
| KP721408.1 | Euclinostomum heterostomum isolate 349/13_6 cytochrome oxidase subunit I (COI) gene, partial cds; mitochondrial | Italy |
